# Impacts of visual neurorehabilitation on autistic children

**DOI:** 10.1192/j.eurpsy.2023.725

**Published:** 2023-07-19

**Authors:** D. A. Marinho, D. R. M. Avejonas, L. Rhein

**Affiliations:** Rehabilitation Sciences, University of São Paulo, Niterói, Brazil

## Abstract

**Introduction:**

Children with Autism Spectrum Disorder (ASD) have a qualitative deficit in social interaction that can be manifested by two of the following characteristics: a) deficits in the use of non-verbal behaviors, such as eye contact, facial expressions, body postures and gestures used to regulate the social interaction; b) inability to develop peer relationships in an adequate manner compatible with their level of development; c) absence of the spontaneous tendency to share emotions, interests and objects; d) lack of social and emotional reciprocity. Among these characteristics, we can mention the difficulty in establishing and maintaining eye contact as one of the points that make it more difficult to develop important skills for learning in general.

**Objectives:**

The present study aims to investigate the impacts of visual neurorehabilitation on autistic children.

**Methods:**

The online questionnaire based on the emotional and functional development scale (FEDQ) of the DIR/Floortime model was distributed to parents and professionals as of October 15, 2022. To date, the Visual Contact Protocol has been applied to 34 children.

**Results:**

The protocol has been applied to 34 children so far.The collection suggests that when we favor the visual contact of the autistic, the motor, cognitive, linguistic, emotional and especially social learning prove to be facilitated

**Conclusions:**

Discussion

Vision is the master of all the senses. When the child is born, his visual ability is very limited. As she grows functional visual skills (HVF) develop. When we talk about the autistic child, these abilities may not be well defined.The collection suggests that when we favor the visual contact of the autistic, the motor, cognitive, linguistic, emotional and especially social learning prove to be facilitated. When observing the children in speech therapy, a better engagement was verified during the execution of the activities suggested by the therapist. Vision is a learned process that adds meaning to what is seen. There is still a lot of research to be done, but by providing the possibility of visual screening to the autistic child, we give them the opportunity to explore the world, get to know and recognize the environments that surround them and consequently improve their learning.Keywords: autism, visual neurorehabilitation.

**Disclosure of Interest:**

None Declared

